# Editorial: Biomedical applications of water-soluble synthetic polymers

**DOI:** 10.3389/fbioe.2023.1235931

**Published:** 2023-06-13

**Authors:** Shuai Liang, Fei Xu, Jiang Wu, Xudong Deng

**Affiliations:** ^1^ Saint-Gobain Research North America, Northborough, MA, United States; ^2^ Department of Chemical Engineering, McMaster University, Hamilton, ON, Canada; ^3^ School of Pharmaceutical Sciences, Wenzhou Medical University, Wenzhou, China; ^4^ School of Life Sciences, Northwestern Polytechnical University, Xi’an, China

**Keywords:** synthetic polymers, hydrogel, biomedical, interpenetrating polymer network (IPN), lubricant

In recent decades, the fusion of polymer science and biomedical research has led to groundbreaking advancements, opening up numerous opportunities to enhance human health and medical interventions. Within the realm of biomedical applications, water-soluble synthetic polymers have become essential tools, fundamentally transforming drug delivery systems, tissue engineering, diagnostics, regenerative medicine, and more. These polymers exhibit a distinctive set of attributes, including biocompatibility, adjustable degradation rates, controlled release mechanisms, and the capacity to encapsulate and safeguard delicate biomolecules. Such characteristics render them remarkably adaptable and ideally suited for a diverse array of therapeutic and diagnostic approaches.

In this Research Topic, we have gathered a few original research articles which highlight the potential of water-soluble synthetic polymers in addressing critical biomedical challenges. Below is a brief summary of published papers in this Research Topic.

Osteosarcoma is a long-term threat to human health, especially for children and adolescents. However, common clinical therapies have the disadvantages of severe side effects and poor prognosis. For example, different types of bone defects may exist after osteosarcoma resection. Therefore, effective treatment of osteosarcoma combining tumor-removing and bone regeneration is urgently required. Based on the thermal sensitivity of tumors, magnetic hyperthermia with mild side effects has received much attention from scholars. Shi et al. designed a 3D printable magnetic hydrogel composed of water-soluble polymer gelatin methacrylate (GelMA) and MeCFO (cobalt ferrite surface-functionalized with methacrylate groups) nanoparticles for osteosarcoma treatment. The MeCFO/GelMA hydrogel has high porosity and swelling ability, indicating that the hydrogel possesses sufficient space, good hydrophilicity, and good compatibility for normal cell growth. MeCFO at 50 μg/mL could not only decrease the cell activity of osteosarcoma cells, but also promote the osteogenic differentiation of bone marrow derived mesenchymal stem cells (BMSCs). Furthermore, the hydrogel with shear thinning property is suitable for serving as bioprinting ink to produce the desired structures using a 3D printer. Therefore, MeCFO/GelMA hydrogel with potential antitumor and bone reconstruction functions is a promising therapeutic strategy after osteosarcoma resection.

Ajaz et al. developed a pH-responsive semi-interpenetrating polymer network (semi-IPN) to address the absorption and stomach upset issues associated with Cetirizine HCl (CTZ HCl). They utilized itaconic acid and acrylamide to construct the polymer matrix through free radical polymerization. Aloe vera, a natural polymer known for its high-water absorbing capacity and ease of modification, was chosen as a key component for the pH-sensitive semi-IPN hydrogel formulation. The resulting hydrogel successfully incorporated CTZ HCl without any negative interactions. The researchers aimed to achieve site-specific delivery of CTZ HCl using the pH-responsive swelling behavior of the semi-IPN hydrogel. Extensive characterization of the developed networks was conducted *in vitro* and *in vivo*. The swelling behavior of the hydrogels was assessed by immersing them in buffer solutions at different pH levels. Sol-gel analysis was performed to determine the non-crosslinked polymeric content. Characterization techniques were used to study the solid state of CTZ HCl and the semi-IPN hydrogels. The drug content was estimated by extracting the dried discs with CTZ HCl and measuring the cumulative drug release. Furthermore, *in vitro* drug release experiments were conducted at pH 1.2 and pH 7.4, and the release mechanism was evaluated using the Korsmeyer-peppas model. Toxicity testing was performed according to fixed dose guideline number 420, and the animals were monitored for 14 days, followed by organ staining for tissue analysis. The results showed that aloe vera significantly increased the swelling index of the semi-IPN hydrogels, and the developed formulation can release a limited amount CTZ HCl in low pH while achieving maximum release in a controlled manner at pH 7.4. Blood analysis and histopathological examination of vital organs indicated no abnormalities or adverse effects.

Surgical wounds might cause long-term pain or potential chronic inflammation, particularly for women who have post-cesarean section scar diverticulum (PCSD). Though conventional treatment such as the hysteroscopic electrocauterization technique has been applied to remove scars, the complex of process and disinfection step are still stressful and painful for most people. Gao et al. developed a water-based lubricant composed of polyethylene glycol (PEG) and chitosan (CS) that was prepared in one-step via ultrasonic blending without any pre- or post-treatment. It was proved that the PEG/CS composite lubricant exhibited excellent thermal stability, high cell compatibility and decent tribological results. *In vivo* experiments were also conducted to confirm the efficacy of the PEG/CS composite lubricant for improving wound healing. Interestingly, the PEG/CS lubricant can enter the wound quickly where CS promotes wound healing process while PEG contributes to tissue regeneration, resulting an accelerated wound closure. Given that both PEG and CS have been widely explored for commercialization in medical products, this PEG/CS composite lubricant show a great potential in clinical use and biomedical applications that is not only limited to PCSD treatment but also applicable for other open wound treatments.

Inflammation and tissue regeneration insufficiency are some of the major factors hindering the healing of chronic wounds. Immunity response and immunomodulatory play essential roles in tissues during wound healing and tissue regeneration. As such, the processes of tissue healing and regeneration can be regulated via modulating immune cell phenotypes, such as macrophages. Sheng et al. synthesized a water-soluble phosphocreatine-grafted methacryloyl chitosan (CSMP) through a one-step lyophilization method, followed by the fabrication of CSMP hydrogel with a photocrosslinked method. It was proved that CSMP hydrogel possessed satisfactory water uptake and compressive strength. Besides, the CSMP hydrogel inhibited the expression of inflammatory factors such as interleukin-1β (IL-1β), IL-6, IL-12, and tumor necrosis factor-α (TNF-α) in an *in vitro* study cocultured with pro-inflammatory factors in pre-treated bone marrow-derived macrophages. The mRNA sequencing results showed that the CSMP hydrogel might inhibit the macrophage’ M1 type polarization through the NF-κB signaling pathway. Overall, the CSMP hydrogel showed great potential for wound defect healing in clinics.

In summary, the compilation of articles in this Research Topic showcases the progress made in the field of biomedical applications of water-soluble synthetic polymers. These polymers are poised to further advance their reach and importance in the realm of biomedicine.

